# Evaluating the impact of needs assessment models on autistic children’s participation in the habilitation process: protocol for a prospective observational study

**DOI:** 10.1136/bmjopen-2024-089135

**Published:** 2024-11-01

**Authors:** Lars-Olov Lundqvist, Linda Sjödin, Evelina Karvonen, Susann Arnell

**Affiliations:** 1 Örebro University, Orebro, Sweden; 2 CHILD research group, Jönköping University, Jonkoping, Sweden; 3 Habilitation Centre, Region Jönköping County, Jonkoping, Jönköping, Sweden; 4 Child and Youth Habilitation Center, Region Örebro län, Orebro, Örebro, Sweden

**Keywords:** Patient Participation, Adolescents, Child, Disabled Persons

## Abstract

**Introduction:**

The rising prevalence of autism spectrum disorder (ASD) among children poses significant challenges for healthcare services. Research has underscored the crucial role of children’s involvement in their healthcare. However, due to the intricate nature of ASD, marked by communication and social interaction differences, healthcare providers face challenges in tailoring their services to accommodate these children. This project aims to explore the impact of two distinct needs assessment models on children’s participation in the needs assessment process.

**Methods and analysis:**

We will conduct a prospective observational study comparing responses from children subjected to two different needs assessment procedures: survey-based and meeting-based. Supplementary data will be collected from the children’s parents/guardians and healthcare professionals. Data collection methods will include questionnaires, interviews and document analysis of individual habilitation plans. We aim to recruit 120 children aged 7–17 diagnosed with ASD but without intellectual disability, with 60 undergoing the survey-based needs assessment and 60 undergoing the meeting-based assessment. The primary outcome measure will be the perception of participation in the needs assessment procedure. Secondary outcomes will include the children’s quality of life and mental health; the parents’ knowledge of their child’s strengths, abilities and special needs; and the parents’ perception of the quality of collaboration with the healthcare team.

**Ethics and dissemination:**

The study received ethics approval from the Swedish Ethical Review Authority on 4 March 2024 (reference number 2024-00227-01). All children and their caregivers will receive both verbal and written information about the study before being asked to provide written informed consent to participate. The findings will be disseminated through publication in peer-reviewed journals and presentation at conferences. Additionally, a popular science report summarising the data and its interpretation will be published.

**Trial registration number:**

NCT06381856.

**STRENGTHS AND LIMITATIONS OF THIS STUDY**

- User involvement will be a key aspect.
- The study will contribute to our understanding of how to enhance the participation of autistic children and adolescents in their healthcare.
- Mixed methods will be used, analysing quantitative and qualitative data from the participating children, parents/guardians and healthcare professionals.
- This will be a non-randomised observational study with limited possibility for causal inferences.
- The study will focus on children with autism spectrum disorder, so the findings may not be generalisable to children with other disabilities.

## Introduction

 Autism spectrum disorder (ASD) presents as a multifaceted neuropsychiatric developmental condition characterised by challenges in social communication along with restricted, repetitive and stereotyped behaviours and interests.^1^ The prevalence of ASD in children varies across studies, with a global estimate of approximately 1%.^2^ Notably, Sweden exhibits a higher prevalence, around 1.6%, ranking among the highest worldwide when assessed using standardised diagnostic criteria such as International Classification of Diseases, Tenth Revision; Diagnostic and Statistical Manual of Mental Disorders, Fourth Edition; or Autism Diagnostic Observation Schedule.^2 3^ Moreover, there has been a notable surge in autism diagnoses globally over recent decades,^4^ with Sweden experiencing a particularly substantial increase.^5^

Sweden has complementary healthcare for all children, and this increase in ASD diagnoses among children poses a significant challenge to the healthcare providers tasked with meeting these children’s specialised needs, particularly those in Child and Youth Habilitation Centres (CYHCs). Central to this challenge is ensuring that CYHCs can effectively address these needs within a reasonable timeframe.

Research has underscored the importance of actively involving children as participants in their habilitation process.^6^ Participation entails two key aspects: first, the child’s attendance, colloquially referred to as ‘being there’, and second, their active engagement and involvement while ‘being there’.^7^ However, a notable gap exists in our understanding of the optimal level of participation for children, especially concerning the mapping of needs and prioritisation of interventions. This gap is particularly pronounced for children with autism, who often deal with challenges in social interaction and communication while also exhibiting repetitive behaviours and narrow interests. These traits can impede the child’s ability to comprehend and engage with topics outside their own interests, complicating their participation in various activities. Consequently, CYHCs are continually refining their processes to enhance these children’s functioning and overall well-being. The initial reception at the CYHC plays a crucial role in this process. However, the degree of a child’s participation in this initial phase remains a subject of inquiry, especially concerning needs assessment and intervention prioritisation.

Enhanced participation of children is also linked to improved mental health outcomes^8^ and quality of life (QoL),^9^ which is particularly significant given the elevated prevalence of co-occurring mental health issues among individuals with autism.^10^,^12^ Thus, it is pertinent to explore the relationship between participation and mental health outcomes for children and adolescents with autism, especially within the context of the initial reception process at CYHCs. While existing research on the impact of autistic children’s participation on mental health is limited, studies involving children with other disabilities suggest a positive correlation.^13^ Thus, there is a need to shed light on the intricate interplay between participation, mental health outcomes and the efficacy of innovative assessment models in meeting the diverse needs of children and adolescents with autism.

### The present study

Given the arguments mentioned above, the present study aims to address these gaps by examining the relationship between participation, mental health outcomes and the efficacy of a new needs assessment model at a CYHC in central Sweden. This model emphasises increased child involvement in needs assessment and goal-setting processes, which could result in the development of more personalised and meaningful intervention plans for the children.

The current survey-based needs assessment model offers a significant advantage in shorter waiting times. However, it comes with a drawback: children with multiple needs receive several interventions simultaneously. This results in a slower and more fragmented habilitation process, often leading to lower participation evaluations in patient surveys. In response to these challenges, the CYHC is implementing a novel needs assessment procedure tailored for the intake of children diagnosed with ASD. The primary purpose of this new assessment model is to enhance the child’s participation in both assessment and goal-setting processes. By doing so, it aims to more effectively identify and prioritise the child’s needs, facilitating a prompt and targeted intervention. Following this initial intervention, additional interventions can be offered to address less prioritised needs, ensuring a comprehensive approach to the child’s care.

In this updated approach, the child and their parent are invited to an initial personal meeting where they receive essential information about the CYHC, engage in discussions about the child’s diagnosis and conduct a preliminary needs assessment. Following this, the child and parent participate in four additional personal meetings to delve deeper into the child’s needs and to establish clear goals that are meaningful to the child.

Because the child’s participation in the needs assessment is an important component of the new reception model, the abovementioned previous research suggests potential effects on the child’s mental health. It is therefore of interest to study the connection between level of participation and mental health, both in the child and in the parents. Another question that is raised in the project is whether by having the parent present at the meeting, the new needs assessment model will lead to the parent changing their view on their child’s ability to act. If the parent perceives their child as competent in the meeting with professionals, this may have a beneficial effect on the child’s self-confidence, which in turn has significance for the child’s participation and mental health.

The interventions at the CYHC are tailored to each child’s specific needs through collaborative intervention planning involving both families and healthcare professionals. Central to this process is the acknowledgement of the child’s perspective and ensuring their voice is heard. The study seeks to explore whether increased child participation correlates with the formulation of more individual-specific goals in the children’s individual habilitation plans (IHPs). These plans function not only as documents but also as dynamic processes, providing guidance to professionals and families in effectively addressing the child’s requirements. In summary, this research endeavours to shed light on the intricate interplay between participation, mental health outcomes and the efficacy of innovative assessment models in meeting the needs of children and adolescents with autism.

### Aim

The primary aim is to investigate the effect of two needs assessment models on children’s participation in the needs assessment process from the child’s, parent’s and professional’s perspective. The secondary aim is to investigate the association of perceived participation with mental health, QoL and formulation of goals in the child’s IHP.

## Methods and analysis

### Study design

The study is a prospective observational study comparing responses from children and parents who underwent a survey-based assessment procedure to responses from those who instead had a meeting-based assessment procedure.

### Participants and sample size

To be eligible for participation in the study, a child or adolescent must be aged 7–17 with a diagnosis of ASD without intellectual disability. No other exclusion criteria will be applied. A power analysis was conducted based on results obtained from pilot testing of items in the Child Involvement Questionnaire (the primary outcome). This analysis determined that 60 children would be required in each group, totalling 120 children, to detect a moderate effect size difference between groups (Cohen’s d=0.5) with 80% power and a 5% alpha level. Since the main focus of the study is on children’s participation, no power calculation was conducted for the number of parents or staff involved. Given an expected intake of eligible children ranging from 300 to 400 per year at the CYHC and assuming a response rate of about 30% (based on experience from previous studies at the CYHC), the data collection period is estimated to be approximately 1 year.

### The needs assessments procedures

#### Survey-based needs assessment

In the survey-based assessment, families are given comprehensive information about the CYHC along with questionnaires which are to be completed and then returned for assessment by a team of professionals. The child is provided with a questionnaire designed to explore areas of daily challenges and areas where assistance is needed, covering topics such as sleep patterns, stress levels, worries, personal hygiene and time management skills. Additionally, the child is asked if they would like more information about their autism diagnosis. Parents are provided with a comprehensive questionnaire that delves into various aspects of their child’s well-being, including sleep patterns, stress levels, worries, eating habits, personal hygiene, time management skills, social interaction and communication abilities. Additionally, parents are given the opportunity to express whether they require further information regarding autism diagnosis or assistance from family and community support networks and to identify the two most pressing areas for intervention.

Based on the responses on the questionnaires and information from the child’s medical record, the team of professionals prioritise and recommend initial treatment options and place the child on a waiting list for the appropriate specialist.

#### Meeting-based needs assessment

In the meeting-based assessment, families are provided with comprehensive information about the CYHC and then contacted to schedule four meetings. These meetings take place at the CYHC and involve the child, one parent and a healthcare professional such as an occupational therapist or a special needs educator. Depending on the child’s age and the arrangements made, the parent may participate throughout the entire meeting or during specific segments.

The purpose of the first meeting is to establish a connection with the therapist, to create an alliance and to allow the child to acclimate to the new environment, understand the diagnosis, feel heard and leave with a positive experience. The second meeting delves deeper into the child’s experience and preferences for meetings, necessary adaptations and activities. In the third meeting, the therapist provides insight into autism and its impact on brain function and then engages the child in discussion about their experience of stress and of maintaining energy balance over time. In line with these discussions, the therapist may also suggest activities aimed at promoting overall well-being. The fourth meeting focuses on summarising the child’s autism profile, identifying their needs and prioritising treatments based on appropriateness rather than simply availability of professionals. Each meeting typically lasts between 30 and 45 min.

### Data collection and instruments

**Figure 1:**
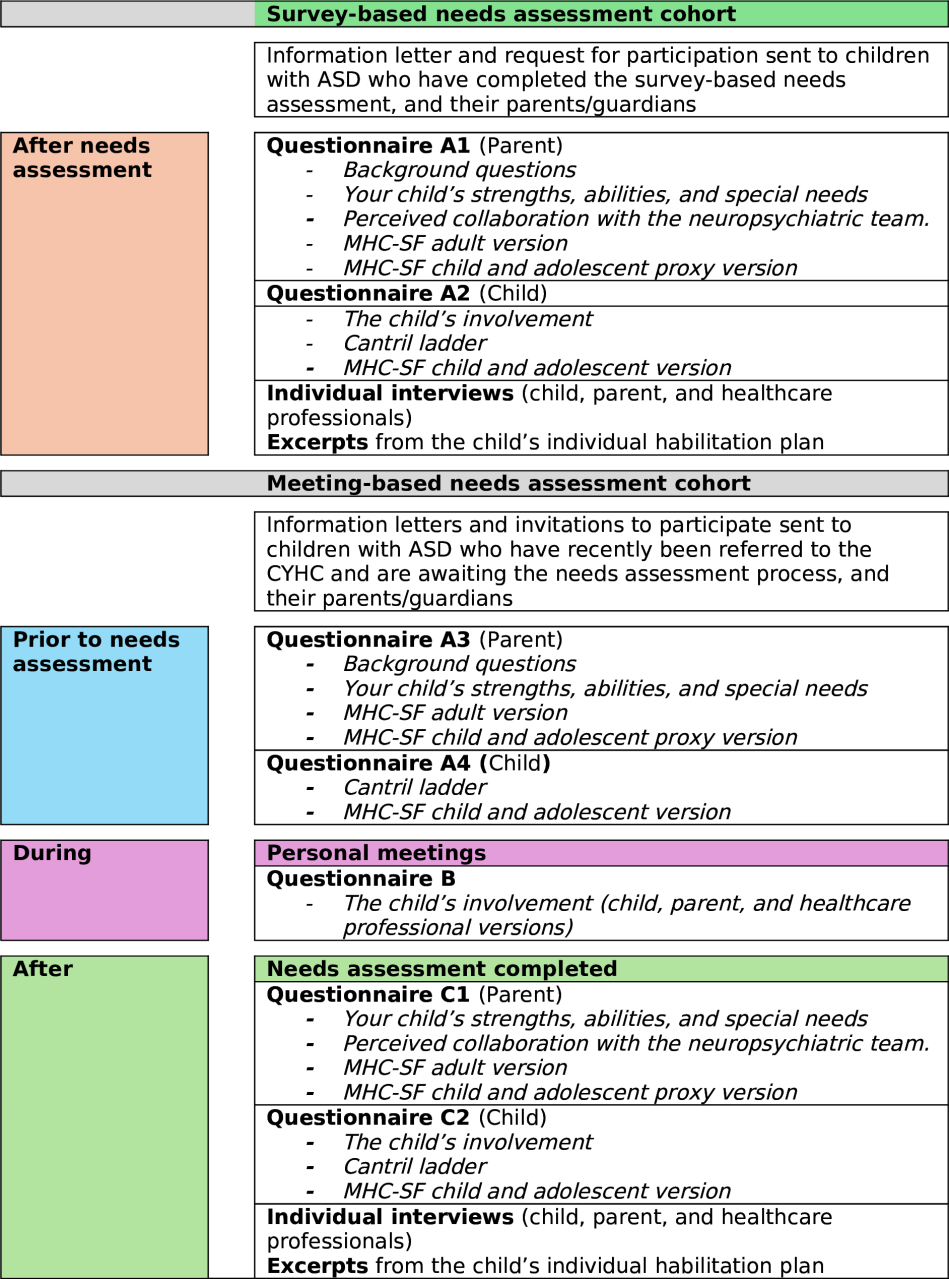
Flowchart of the project process. ASD, autism spectrum disorder; CYHC, Child and Youth Habilitation Centres; MHC-SF, Mental Health Continuum-Short Form.

Patients will be invited to participate consecutively as they are diagnosed and registered at the CYHC. Data will be collected using questionnaires, interviews and texts from the children’s IHPs. Figure 1 summarises how and to whom the questionnaires will be distributed. The questionnaires will appear in different variants to suit the two cohorts as well as the person answering (child, parent or health professional). Questionnaires will be compiled in a booklet to make it easier to fill them in. The inclusion of patients is planned to begin in June 2024, and the expected completion of the study is December 2025.

#### Measures

*Background questions*. A set of eight sociodemographic questions concerning the child and the family.

*Your child’s strengths, abilities and special needs*. This questionnaire, adapted from the Family Outcome Survey,^14^ aims to gather insights into parents’ understanding of their child’s profile. Parents respond on a 5-point Likert scale ranging from ‘Nothing/Never’ to ‘Everything/Always’. Two versions are available: one with four questions for use before needs assessment meetings and another with five questions for use after the final meeting or for the survey-based group after the needs assessment completion. This questionnaire is completed solely by parents.

*Perceived collaboration with the neuropsychiatric team*. Comprising five questions, this questionnaire assesses the family’s interaction with the neuropsychiatric team at the CYHC and the child’s involvement during these interactions. Responses are provided on a 5-point Likert scale. This questionnaire is completed solely by parents.

*The Mental Health Continuum-Short Form(MHC-SF)*.^15^ This questionnaire consists of 14 questions rated on a 6-point Likert scale, measuring mental health across three domains: emotional, psychological and social well-being. The MHC-SF has been validated from 7 years of age in Portuguese speaking children^16^ and from 12 years of age in Swedish speaking children.^17^ In the present project, three versions are available: one where the child self-reports, one where the parent self-reports and one where the parent assesses the child’s mental health (proxy assessment).

*Cantril ladder*.^18^ This tool evaluates QoL using a visual representation of a ladder numbered from 0 (lowest QoL) to 10 (highest QoL) in a valid and reliable manner.^19^ It is completed solely by the children.

*The child’s involvement*. This questionnaire contains seven to nine questions regarding participation and engagement during needs assessment meetings. It is available in three versions: one for the child, one for the parent and one for the healthcare professional. Each version has two variants tailored for regular meetings and the closing meeting. Responses are rated on a 4-point Likert scale ranging from ‘Not at all’ to ‘Very much’.

### Qualitative interviews

Following the establishment of the child’s IHP, individual interviews will be conducted with a selection of children, parents and healthcare professionals involved in the care planning discussions from both cohorts. These interviews will explore their perspectives and insights on the concept of participation, the child’s involvement in the actual needs assessment process and strategies for promoting participation. This includes discussing the specific support provided by healthcare professionals to enhance participation.

### Data management

Each of the participating children will be assigned a unique identifying number that will be used to establish a link between the various data collection forms, including parental and healthcare professionals’ questionnaires. All collected data will be stored pseudonymously in a dedicated research data storage area designed to meet stringent requirements for handling sensitive personal information. This storage solution is integrated into a centralised file server system, ensuring redundancy, backup and secure access controls. Participant confidentiality will be rigorously upheld, with stringent measures in place to prevent unauthorised access to the data.

### Data analysis plan

Descriptive statistics will be used to summarise the sociodemographic characteristics of the children and their families. The questionnaires will be analysed using inferential statistical analysis such as t-test and analysis of variance, whereas associations between variables will be analysed by correlation and multiple regression. Multiple imputation will be used to handle missing data. The individual interviews will be transcribed verbatim and then analysed with inductive content analysis as described by Graneheim and Lundman.^20^ Document analysis of excerpts of the children’s IHP’s will be performed with quantitative content analysis according to Krippendorf^21^ and qualitative content analysis as described by Graneheim and Lundman,^20^ with a focus on descriptions of formulated goals. Trial findings will be reported according to CONSORT 2010 guidelines.^22^ The SPIRIT reporting was used for this submission.^23^

### Patient and public involvement

The study protocol has been reviewed for input by a reference group established for this purpose, comprising a parent of autistic children, professionals working with children with ASD, a representative from the management of the CYHC and researchers. Publications and presentations involving data generated from this project will be interpreted and disseminated by the reference group and by the child and adolescents’ advisory group at the CYHC. Research institutions as well as non-governmental organisations targeting children with ASD, such as Autism Sweden, the Swedish National Association Attention and the Swedish National Society of Autism, will be invited to work together to translate the research into benefits for the autistic community, including awareness raising and capacity building.

### Ethics and dissemination

Ethical approval for the study was provided by the Swedish Ethical Review Authority (reference number 2024-00227-01). The study protocol adheres to the ethical guidelines outlined in the Declaration of Helsinki.^24^ Participants will be provided with comprehensive information about the study, including details about the consent process, the voluntary nature of participation and the option to withdraw without needing to provide a reason. Written consent will then be obtained (online supplemental appendix 1). Results of the study will be disseminated via presentations at scientific conferences and publication in open access, peer-reviewed journals. Participants will receive prompt updates on the study’s findings once the results become available. Additionally, the study’s conclusions will be shared with members of the autistic community. To ensure wider accessibility, a popular science report summarising both the data and its interpretation will be published in Swedish. This will cater to professionals working with children with ASD and anyone else interested in the study’s topic.

## Discussion

This article outlines the rationale and design of the study, detailing the recruitment process, the content of the needs assessment procedures, the outcome measures and the quantitative and qualitative data analyses. If successful, the study will yield insights into the effects and efficacy of enhancing the participation of autistic children in the habilitation process. It will also shed light on the obstacles and enabling factors for implementing needs assessment procedures in healthcare settings.

The strengths of the study lie in its user involvement in both design and result interpretation, the provision of detailed profiles for individual children and families and the utilisation of a mixed-methods approach incorporating quantitative and qualitative data from children, parents and healthcare professionals. However, a methodological limitation is the observational design, which restricts the ability to draw causal inferences. Moreover, as the study will be conducted in a single region in Sweden with a focus on children with ASD, the findings may not be directly generalisable to children in other countries or with different disabilities. Nonetheless, it is reasonable to infer that other autistic children may share similar needs and could derive comparable benefits from enhanced participation in the habilitation process. Finally, children’s understanding of participation is likely to vary with age, with older children demonstrating more advanced reasoning. However, the instruments used for this study have already been tested in a previous study involving children within the same age range. To further capture the children’s voices and perceptions, we will conduct interviews, allowing them to express their own views on what participation means to them and how they felt they were able to participate in the assessment process.

In conclusion, if the study achieves its objectives, it has the potential to enhance children’s participation in their care, thereby potentially improving their mental health and QoL. This, in turn, could lead to reduced need for support and service from healthcare providers.

## supplementary material

10.1136/bmjopen-2024-089135 online supplemental file 1
