# Supplementary material for: Evaluating the impact of needs assessment models on autistic children’s participation in the habilitation process: protocol for a prospective observational study
Source: BMJ Open. 2024 Nov 1;14(10):e089135. doi: 10.1136/bmjopen-2024-089135 (PMC11529756; doi:10.1136/bmjopen-2024-089135)
Supplement: online supplemental file 1 [file bmjopen-14-10-s001.pdf]

Appendix 1. Informed consent materials

Translations from the original consent forms in Swedish.

Children's form:

Consent to participate in the project: Participation of autistic children and adolescents in the habilitation process

I have received oral and/or written information about the project and have had the opportunity to ask questions. I get to keep the written information..

I agree to participate in the project and to my personal data being processed as described in the research person information.

Place and date	The child's signature
	Name clarification

After signing, post the form in the enclosed reply envelope.

Parent’s form:

Consent to participate in the project: Participation of autistic children and adolescents in the habilitation process

I/we have received written information about the project and have had the opportunity to ask questions. I/we may retain the written information.

I/we agree that I/we and ____________________________ (child's name) and date of birth ____________________ are included in the project and that information about me/us and my/our child is processed in the manner described in the research person information.

Address: ____

____

Telephone: ______________________________

If you are two parents/guradians, both must sign

Place and date	Signature parent 1
	Name clarification

Place and date	Signature parent 2
	Name clarification

After signing, post the form in the enclosed reply envelope.

Staff’s form:

Consent to participate in the project: Participation of autistic children and adolescents in the habilitation process

I have received oral and written information about the project and have had the opportunity to ask questions. I may retain the written information.

I agree to participate in the project and to my personal data being processed as described in the research person information.

Address: ____

____

Telephone: ______________________________

Place and date	Signature
	Name clarification

After signing, post the form in the enclosed reply envelope.
