# Supplementary material for: Evaluating the impact of needs assessment models on autistic children’s participation in the habilitation process: protocol for a prospective observational study
Source: BMJ Open. 2024 Nov 1;14(10):e089135. doi: 10.1136/bmjopen-2024-089135 (PMC11529756; doi:10.1136/bmjopen-2024-089135)
Supplement: Supplementary file 2 [file bmjopen-2024-089135.reviewer_comments.pdf]

PEER REVIEW HISTORY

BMJ Open publishes all reviews undertaken for accepted manuscripts. Reviewers are asked to complete a checklist review form and are provided with free text boxes to elaborate on their assessment. These free text comments are reproduced below.

ARTICLE DETAILS

Title (Provisional)

Evaluating the impact of needs assessment models on autistic children's participation in the habilitation process: protocol for a prospective observational study

Authors

Lundqvist, Lars-Olov; Sjödin, Linda; Karvonen, Evelina; Arnell, Susann

---

VERSION 1 - REVIEW

**Reviewer**	**1**
**Name**	**Thabrew, Hiran**
**Affiliation**	**University of Auckland, Psychological Medicine**
**Date**	**18-Aug-2024**
**COI**	**None**

Dear authors

It was a pleasure to review your protocol submitted to BMJ Open.

First, allow me to commend you on undertaking a project to improve the ways in which the voice of children and young people are included in care planning. There is currently limited research about how best to do so, and I'm sure your results will be of interest to clinicians around the world.

Your manuscript is well-written and information is presented in keeping with SPIRIT guidelines. While I do not have any major concerns, I do offer the following suggestions for improvement and/or clarification within the manuscript:

1. It is unclear at what stage of the assessment process children will be invited to participate. Just after receiving a formal diagnosis? At any time, and possibly years later? It would be good to make this easier for readers to understand.

2. While a statement is made on P7 that there are 'no exlusion criteria', it seems that having an intellectual disability is indeed a criterion for exclusion. So, perhaps rephrase as 'no other exclusion criteria'?

3. The age range for participants is rather large. Given differences in cognitive ability (especially abstract thinking) that might influence ability to understand and participate in decision making, was any thought given to some kind of subgroup analysis (say under and over 12s)? I realise this has an impact on the power calculation for the study and that your proposal has already received ethics committee approval. However, it might be worth thinking about at this stage, lest significant cognitive variability among your cohort dilutes any possible observed effects. Even if you do not wish to change your protocol, some discussion of this issue is warranted.

4. For what ages has the MHC-SF been validated? Could psychometric properties for this and other questionnaires for which they are available please be included?

5. Are the health professionals who will be interviewed those who have been involved in the care planning discussions or those who subsequently provide treatment. This is not clear.

6. Could a table or additional text be included to make more explicit the connection between research Qs/aims , measures and methods by which that data will be analyed? Given that you have three types of participant and multiple qualitative and quantitative analyses planned, this is not currently obvious.

I hope these suggestions are of help and wish you all the best with revising your manuscript prior to publication.

Reviewer **2**

Name **Tromans, Samuel**

Affiliation **University of Leicester, Department of Population Health Sciences**

Date **20-Aug-2024**

COI **I have been an co-applicant on funding from the Wellcome Trust relating to autism, as well as received NIHR research support for autism related research. I am also an editorial board member of the journal Advances in Autism. I sit on the executive committee of the Neurodevelopmental Psychiatry Special Interest Group at the Royal College of Psychiatrists. I have also recently been a member of an expert reference group group led by the autism charity Autistica.**

• Thank you for inviting me to review this protocol manuscript relating to the participation of autistic children and adolescents in the habilitation process, which was a pleasure to read. The manuscript is clearly written and presented. I have some further comments detailed below, intended to further enhance what is already a good manuscript.

• Line 67 – Consider using the term 'autism' rather than 'autism spectrum disorder', as the former term is more widely accepted by the autistic community

• Line 73 – Technically the ADOS is not diagnostic criteria – a clinical diagnosis should not be based on ADOS findings alone, though in research practice ADOS alone is sometimes used as a proxy measure of autism

• Line 99 – Identity-first language is generally preferred by the autistic community when referencing autistic people (i.e., 'autistic individuals' rather than 'individuals with autism.')

• Lines 142-143 – Can the authors give any specific examples of how acknowledgement and recognition of the child's perspective is ensured?

• Lines 164 – Can the authors give a rationale for the exclusion of children with co-occurring intellectual disability?

• Line 182 – The sentence should probably read 'more information about their autism diagnosis.'

• Lines 218-244 – When discussing the various study measures, the authors should report details about previous validation studies for all measures, whereas they have only done this in the case of the Cantril ladder.

• Lines 285-293 – Were any autistic children asked for their views in the participant and public involvement process?

• Finally, I would recommend a statistical reviewer to review the power calculation and various statistical tests proposed.

VERSION 1 - AUTHOR RESPONSE

Please find the responses to the reviewers' comments in the attached document

Reviewer 1 comments:

It was a pleasure to review your protocol submitted to BMJ Open.

First, allow me to commend you on undertaking a project to improve the ways in which the voice of children and young people are included in care planning. There is currently limited research about how best to do so, and I'm sure your results will be of interest to clinicians around the world.

Your manuscript is well-written and information is presented in keeping with SPIRIT guidelines. While I do not have any major concerns, I do offer the following suggestions for improvement and/or clarification within the manuscript:

Authors´response. Thanks for the appreciation! It is indeed an important area to study.

R1.1. It is unclear at what stage of the assessment process children will be invited to participate. Just after receiving a formal diagnosis? At any time, and possibly years later? It would be good to make this easier for readers to understand.

Authors´response. The children will be invited as they are diagnosed and registered at the habilitation centre. A text to clarify this has been added on line 215.

R1.2. While a statement is made on P7 that there are 'no exlusion criteria', it seems that having an intellectual disability is indeed a criterion for exclusion. So, perhaps rephrase as 'no other exclusion criteria'?

Authors´response. Thank you for the comment. We reasoned that by including only children with autism who do not have an intellectual disability, the group inherently excludes individuals with intellectual disabilities. Therefore, there is no need for additional exclusion criteria on that basis. However, as you suggest we have rephrased the following sentence to 'no other exclusion criteria'.

R1.3. The age range for participants is rather large. Given differences in cognitive ability (especially abstract thinking) that might influence ability to understand and participate in decision making, was any thought given to some kind of subgroup analysis (say under and over 12s)? I realise this has an impact on the power calculation for the study and that your proposal has already received ethics committee approval. However, it might be worth thinking about at this stage, lest significant cognitive variability among your cohort dilutes any possible observed effects. Even if you do not wish to change your protocol, some discussion of this issue is warranted.

Authors´response. We appreciate your comment and agree that children's understanding of participation is likely to vary with age, with older children demonstrating more advanced reasoning. However, the instruments used for this study have already been tested in a previous study involving children within the same age range. To further capture the children's voices and perceptions, we will conduct interviews, allowing them to express their own views on what participation means to them and how they felt they were able to participate in the assessment process. A clarifying text has been added in the Discussion section.

R1.4. For what ages has the MHC-SF been validated? Could psychometric properties for this and other questionnaires for which they are available please be included?

Authors´response. Thank you for your insightful comment. MHC-SF has been validated for children down to 7 years of age on a sample of typically developed Portuguese speaking children. As of yet, MHC-SF has been validated for children down to 12 years of age in Sweden which is similar as in the Netherlands and Spain. A clarifying text has been added in the Data collection and instruments section.

R1.5. Are the health professionals who will be interviewed those who have been involved in the care planning discussions or those who subsequently provide treatment. This is not clear.

Authors´response. Yes, that is correct. We have clarified this on line 255

R1.6. Could a table or additional text be included to make more explicit the connection between research Qs/aims , measures and methods by which that data will be analyed? Given that you have three types of participant and multiple qualitative and quantitative analyses planned, this is not currently obvious.

Authors´response. Thank you for your helpful comment. The flowchart in Table 1 is designed to clarify the questions, measures, and methods that will be used throughout the study. We apologize if the current presentation is unclear. While we considered using a CONSORT flowchart, that format is more suited to illustrating the flow of two parallel groups (e.g., intervention and control), whereas our study follows a serial design with two cohorts receiving different treatments sequentially. We acknowledge the limitations of the current flowchart.

I hope these suggestions are of help and wish you all the best with revising your manuscript prior to publication.

Authors´response. Thank you very much for your helpful suggestions and kind wishes. We appreciate your feedback and will carefully consider your input as we work on revising the manuscript.

Reviewer 2 comments:

R2.1 Thank you for inviting me to review this protocol manuscript relating to the participation of autistic children and adolescents in the habilitation process, which was a pleasure to read. The manuscript is clearly written and presented. I have some further comments detailed below, intended to further enhance what is already a good manuscript.

Authors´response. Thank you very much for the encouragement and valuable comments.

R2.2 Line 67 – Consider using the term 'autism' rather than 'autism spectrum disorder', as the former term is more widely accepted by the autistic community

Authors´ response. Thank you for the comment. We acknowledge the use of the term *autism* by the autistic community, however, in the study we are referring to autistic children having a medical diagnosis of ASD, hence the reference to APA, 2013.

3 R2. Line 73 – Technically the ADOS is not diagnostic criteria – a clinical diagnosis should not be based on ADOS findings alone, though in research practice ADOS alone is sometimes used as a proxy measure of autism

Authors´ response. Thank you for your valuable comment. We fully agree that a diagnosis should not rely solely on ADOS, ICD-10, or DSM-IV criteria. We do not intend to suggest otherwise. Our aim in this section is to emphasize the number of children diagnosed according to these specified diagnostic criteria, rather than to imply that any single criterion is sufficient for a comprehensive diagnosis.

R2.4 Line 99 – Identity-first language is generally preferred by the autistic community when referencing autistic people (i.e., 'autistic individuals' rather than 'individuals with autism.')

Authors´ response. Thank you for highlighting this important issue. We recognize and respect the preference for identity-first language within the autistic community. However, given that the primary focus of this study is to evaluate the effectiveness of an intervention, our emphasis is on the methodology rather than the individuals themselves. As such, we have chosen to use person-first language in this context.

R2.5 Lines 142-143 – Can the authors give any specific examples of how acknowledgement and recognition of the child's perspective is ensured?

Authors´ response. A detailed explanation of how the child's perspective is considered is already provided in the 'Meeting Based Needs Assessment' section (lines 195-212). Including this information in the introduction would result in unnecessary duplication.

R2.6 Lines 164 – Can the authors give a rationale for the exclusion of children with co-occurring intellectual disability?

Authors´ response. Thank you very much for your insightful comment. The primary reason for not including children with autism and co-occurring intellectual disabilities is that their care falls outside the scope of the unit involved in this study. Additionally, we determined that we lacked the necessary resources to extend the study to this population. This decision was made purely for practical reasons and is in no way a reflection of the importance of studying this group, which we fully acknowledge as equally, if not more, significant.

R2.7 Line 182 – The sentence should probably read 'more information about their autism diagnosis.'

Authors´ response. Thank you for the perceptive comment. We have revised the text accordingly.

R2.8 Lines 218-244 – When discussing the various study measures, the authors should report details about previous validation studies for all measures, whereas they have only done this in the case of the Cantril ladder.

Authors´ response. Thank you for the comment. We have added validation information on the MHC-SF. None of the other measures have been validated yet.

R2.9 Lines 285-293 – Were any autistic children asked for their views in the participant and public involvement process?

Authors´ response. Yes, autistic children were asked through their parents in the existing reference group and via the so-called "children's council" at the Child and Youth Habilitation Centre.

R2.10 Finally, I would recommend a statistical reviewer to review the power calculation and various statistical tests proposed.

Authors´ response. Thank you for the comment. The research group has the methodological and statistical competence needed in the study. However, to accommodate the suggestion, we have had a professional biostatistician in our organization review the power calculation and our choice of statistical methods.

VERSION 2 - REVIEW

**Reviewer**	**1**
**Name**	**Thabrew, Hiran**
**Affiliation**	**University of Auckland, Psychological Medicine**

Date **10-Oct-2024**

COI

Thank you for addressing my previous queries. I do not have any additional suggestions for the improvement of this paper prior to publication and wish your team all the best with the planned study.
